# The clinical and endoscopic aspects of peptic ulcers secondary to the use of nonsteroidal anti-inflammatory drugs of various origins

**DOI:** 10.11604/pamj.2021.38.170.17325

**Published:** 2021-02-15

**Authors:** Oumar Traoré, Abdoul Salam Diarra, Oumar Kassogué, Tawfiq Abu, Aguissa Maïga, Moussa Kanté

**Affiliations:** 1 Service of Gastro-Enterology, Regional Hospital of Sikasso, Sikasso, Mali,; 2 Reproductive Health Division, Regional Health Direction, Mopti, Mali,; 3 Service of Laboratory, Blood Bank, Regional Hospital of Sikasso, Sikasso, Mali,; 4 Department of Urology, Hassan II University Hospital Center, Fez, Morocco,; 5 Administration Division, Regional Health Direction, Mopti, Mali,; 6 Anesthesia-Reanimation Service, Hospital of Sikasso, Sikasso, Mali

**Keywords:** Non-steroid anti-inflammatory, clinical aspects, endoscopy, gastro-duodenal ulcer

## Abstract

**Introduction:**

currently, the non-steroid anti-inflammatory drugs constitute a veritable object of auto medication throughout the world. The goal of this study was to evaluate the endoscopic and clinical aspects of gastro-duodenal ulcer secondary to taking of non-steroid anti-inflammatory of various sources.

**Methods:**

this was a cross-sectional study which was conducted between July 2016 and December 2017. All adult patients admitted to hospital for clinical symptoms suggestive of gastroduodenal involvement after taking anti-inflammatory drugs and having undergone upper digestive endoscopy were included in this study. Data analysis was done with Epi-info version 7 Software.

**Results:**

a total of 114 patients were included, the mean age was 47.18±26 years with a male predominance (64.9%). Among the patients, only 1.75% had taken a non-steroid anti-inflammatory (NSAIDs) from pharmacy. The NSAIDs used were of different types: diclofenac, aceclofenac, aspirin and non-selective NSAIDs. For each drug used, more than half were derived from the streets. Clinically we noted: the dyspepsia (38.58%), hemorrhages (11.40%), the ulcerous syndrome (77.19%), haematemesis (19.29%), haematemesis associated with melena (37.71%), and the rectorrhagia in 6.14 of cases. The specific endoscopic lesions were bulbar ulcer (45.61%), gastric ulcers (20.17%), antral ulcerations (5.26%) and acute gastritis (9.64%), esophagitis (7.89%), esophageal varices (6.14%), and uncomplicated hiatal hernia in 7.01% of cases.

**Conclusion:**

the serious gastroduodenal lesions observed in this study and due to use of NSAIDs are mainly attributable to unauthorized molecules due to safety concerns. It would be necessary to conduct sensitization days at the community level and in each health facility.

## Introduction

Nonsteroidal anti-inflammatory drugs (NSAIDs), molecules widely used around the world are responsible for many gastrointestinal manifestations whose consequences are a source of high morbidity and mortality in our regions. The illicit proliferation at points of sale of these drugs constitutes a constant danger that seriously threatens the health of users. Thus, symptoms of different endoscopic aspects revealed in our practices at Sikasso Hospital in Mali, must challenge more than ever in the fight against this scourge. Illicit sale and self-medication are behaviors that constitute a real public health problem. The aim of this work was to evaluate the clinical and endoscopic aspects of peptic ulcers secondary to taking NSAIDs from various sources.

## Methods

This was a cross-sectional study that included all adult patients admitted to hospital for clinical symptoms suggestive of gastroduodenal involvement secondary to taking anti-inflammatory drugs and having undergone upper gastrointestinal endoscopy. Patients who have ingested steroids, unidentified molecules and other gastrointestinal drugs for suicidal purposes have not been included. This work was conducted between July 2016 and December 2017 at the Sikasso regional hospital in Mali. The data was collected using a standardized questionnaire and their analysis was done by Epi-info version 7 software.

## Results

A total of 114 patients were involved in our study, of which 64.9% were male. The mean age was 47.18±26 years (range 17 to 89 years). A history of comorbidity was noted in 53 patients (46.49%), drug combinations (corticosteroids, beta-blockers and NSAIDs) in 6 cases (5.26%) and overdose NSAID in 2 cases (1.75 %). The NSAIDs used were of different types: diclofenac (40.35%), the majority (28.07%) coming from “street” or “pharmacy by land”; the aceclofenac (33.33%) of which 18.42% came from the street; aspirin (7.89%), and non-selective NSAIDs (6.14%). The average time between use of these medications and onset of symptoms ranged from 6 hours to 2 days. Clinically, we noted: dyspepsia in 38.58% of our patients; severe haemorrhage in 11.40%; ulcer syndrome in 88 patients (77.19%); hematemesis in 19.29%; haematemesis associated with melena in 37.71%; melena in 14.03% of cases and a rectorrhagia in 6.14%. Endoscopy showed specific lesions such as bulbar ulcer (45.61%), gastric ulcers (20.17%), antral ulcerations (5.26%) and acute gastritis (9.6%) and esophagitis (7.89%). Endoscopy also revealed oesophageal varices (6.14%) and uncomplicated hiatal hernia in 7.01% of cases. The spontaneous cessation of hemorrhages was obtained in 64.91% and bleeding recurrence was observed in 8.77% of cases. Successful adrenalinated serologic endoscopic treatment of sclerosed hemorrhage was administered in 2.63% of cases. We recorded 1 case of death.

## Discussion

Nonsteroidal anti-inflammatory drugs (NSAIDs) pose a real public health problem not only because of their uncontrolled use, but also because of their wide prescription and the seriousness of their undesirable effects. United States and United Kingdom studies have shown that NSAID gastropathy is the most common adverse drug effect in the US and UK. Annual mortality from gastrointestinal complications of these drugs has been estimated at 16,500 deaths [1,2]. The main mechanism responsible for the toxicity of NSAIDs comes from the inhibition of prostaglandin synthesis in the digestive mucosa. The recent discovery of two cyclooxygenase (COX) isoenzymes responsible for the synthesis of prostaglandins has led to the development of drugs that protect the gastroduodenal mucosa. COX-2 is an inducible enzyme that plays a role in inflammation and pain, while COX-1 is an important enzyme in the synthesis of gastro-protective prostaglandins in the stomach and duodenum [3]. Non-selective NSAIDs inhibit both COX-1 and COX-2, resulting in a beneficial effect on inflammation and pain, but potentially harmful in some sites, such as the gastrointestinal tract. The development of highly selective COX-2 inhibitors has been effective in controlling pain and inflammation without causing adverse effects related to COX-2 inhibition. Two highly selective COX-2 inhibitors, celecoxib and rofecoxib, have recently been approved by the International drug control office (OICM) [4].

The clinical manifestations may be of variable intensity, like epigastralgia, nausea, vomiting, dyspepsia, atypical abdominal pain. Sometimes a complication such as upper gastrointestinal bleeding or perforation may be the inaugural symptom. These complications are unpredictable and frightening. The symptomatology sometimes starts immediately after taking the NSAID without warning sign [5]; but most often around the 3^rd^ -4^th^ day. Other times, it is later, manifests itself several days after stopping treatment. In Sikasso (3^rd^ administrative region of Mali) as in many other regions of developing countries, poverty is a factor that has contributed greatly to the high number of “street drugs” still called “pharmacy by land”. In addition, the high cost of certain medicines in pharmacies, the low income of populations and the chronicity of certain diseases contribute to our local populations' use of “pharmacy on land” which is considered by them as “cheaper and quickly accessible”. These medicines sold on the streets kill people, they are fake, badly maintained and mostly expired. They constitute a danger to consumers. Very few studies have been carried out to evaluate the clinical and endoscopic aspects of gastroduodenal involvement related to the consumption of street drugs. This is why our study focused on these aspects related specifically to the consumption of NSAIDs of various origins. In this study, the NSAIDs consumed came in more than half of the cases from “pharmacies on the ground” which also implies a strong self-medication. Nearly 47% of admitted patients had a combination medication, which increases the risk of symptoms and digestive damage.

Clinically, dyspepsia was quite common in our patients with 38.58%. These data are well above those found in the literature despite their frequency [6]. Some authors report that about 20 to 30% of patients on long-term NSAIDs complain of dyspeptic disorders [7,8]. This difference observed in our study compared to the data of the literature could be explained not only by the quality (composition, active principle, preservation mode) of the molecules used but also by the absence of medical prescription (indication according to the field, the ingested dose, setting time and lack of gastric protection). The cases of bleeding observed were serious in 11.40% and this can be explained by the dose of NSAID ingested, the medical history, the advanced age of some patients and by the severity of the digestive lesions due to the absence of gastric protection. Literature reports that the risk of upper gastrointestinal bleeding is multiplied by a factor of 2 to 4 for doses of 75 to 325 mg/day [9] and it is not significantly different according to the dosage form [10]. Given the frequency of low-dose aspirin therapy in Western countries, the number of ulcerative complications related to this type of treatment is considerable. In a recent study in the UK [11], it was similar to that of non-salicylated NSAIDs.

In the Mucosa study of more than 8,000 patients, the association of advanced age, ulcerative or bleeding history, and cardiovascular comorbidity increased the risk of ulcer complications by a factor of 10 compared to a subject exposed to one of these risk factors [12]. The specific endoscopic lesions found in our study were bulbar ulcer (45.61%), gastric ulcers (20.17%), antral ulcerations (5.26%) and acute gastritis in 9.64%. These data corroborate those found in literature in general. Erosive lesions are more often situated in the stomach than in the duodenum. They are constant after acute ingestion of aspirin [13]. In non-salicylated long-term NSAID treatments, the prevalence of these lesions varies from 20 to 80% depending on the populations studied and the molecules consumed [14-16]. Several studies have reported that the presence of erosions increases the subsequent risk of peptic ulcer disease (UGD) [17-19]. Experience shows that digestive tolerance is highly variable from one NSAID to another in the same subject. Symptomatic treatment may also be proposed. Misoprostol is not recommended because it causes digestive side effects such as abdominal pain and diarrhea. Omeprazole whose efficacy has been reported versus placebo [20], ranitidine [21] and misoprostol [22] should be preferred. In our series, none of the patients had taken a gastric protective drug, which contributed greatly to the severity of the symptoms and endoscopic lesions.

## Conclusion

The serious gastroduodenal lesions observed in this study due to the use of NSAIDs are mainly attributable molecules which were not authorized for safety reasons. Young adult males were the most affected. This state of affairs must appeal to all. It would be necessary for our health authorities in collaboration with border countries, the state media, doctors, pharmacists and the trade union of consumers to combine their efforts to raise the awareness of the population and the fight against the proliferation of “pharmacies on the ground”.

### What is known about this topic

- Non-steroid anti-inflammatory drugs can alter the mechanism of defense of mucosa and entrain the occurrence of ulcerous lesions and their complications;
- The illicit proliferation at points of sale of these drugs constitutes a constant danger that seriously threatens the health of users;
- Illicit sale and self-medication are behaviors that constitute a real public health problem.

### What this study adds

- Being the first study on the theme in Mali, it provides for this purpose information contributing to decision-making (communication days for behavioral change at the level of each health facility and the media) on self-medication and illegal sale drugs in general;
- This study also provides an idea of the ampleness of the problem at the local and national stages.
